# Optimization of Water and Nitrogen Application Rates for Synergistic Improvement of Yield and Quality in Solar Greenhouse Cucumber Production on the North China Plain

**DOI:** 10.3390/plants14091285

**Published:** 2025-04-23

**Authors:** Chunting Wang, Xiaoman Qiang, Kai Wang, Huanhuan Li, Xianbo Zhang, Shengxing Liu, Xuewen Gong

**Affiliations:** 1 Institute of Farmland Irrigation, Chinese Academy of Agriculture Sciences, Xinxiang 453002, China; 82101232118@caas.cn (C.W.); lihuanhuan@caas.cn (H.L.); zhangxianbo@caas.cn (X.Z.); liushengxing205@gs.zzu.edu.cn (S.L.); 2 Graduate School of Chinese Academy of Agricultural Sciences, Beijing 100875, China; 3 Henan Province Soil and Fertilizer Station, Zhengzhou 450002, China; tfzbgs701@163.com; 4 School of Water Conservancy, North China University of Water Resources and Electric Power, Zhengzhou 450045, China; gxw068@126.com

**Keywords:** *Cucumis sativus* L., yield, quality, water–nitrogen use fertilizer, yield–quality synergy

## Abstract

To address the scientific challenges of low water–fertilizer use efficiency and the difficulty in achieving the synergistic improvement of the yield and quality in solar greenhouse cucumber production on the North China Plain, this study investigated the effects of varying water and nitrogen supplies on cucumber growth, yields, water–nitrogen use efficiency, and quality. The aim was to establish optimized water and nitrogen management strategies for high-yield, high-quality, and resource-efficient cultivation. A two-factor completely randomized design was implemented, with three irrigation levels (W1: $1.0{\;E}_{p-20}$, W2: $0.75{\;E}_{p-20}$, and W3: $0.5\;E_{p-20}$) based on cumulative pan evaporation and four nitrogen application amounts (N1: 432 kg·ha^−1^, N2: 360 kg·ha^−1^, N3: 288 kg·ha^−1^, N4: 216 kg·ha^−1^). Cucumber growth indicators were observed during the growing season, and the water and nitrogen application rates were scientifically optimized. The results showed that a full water and nitrogen supply enhanced the leaf area index, dry weight accumulation, and yield. Moderate water and nitrogen savings had a minimal impact on plant growth and production while significantly improving the water and fertilizer use efficiency. Using principal component analysis to comprehensively evaluate the cucumber quality, it was found that the irrigation amount had a significant impact on quality, with the quality improving as the irrigation amount decreased. By employing a regression formula and spatial analysis methods, this study optimized the water and nitrogen application rates with the goals of maximizing the cucumber yield, water–nitrogen efficiency, and quality. For spring cucumbers, the recommended combination is an irrigation amount of 225~240 mm and a nitrogen application amount of 350~380 kg·ha^−1^. For autumn cucumbers, the recommended combination is an irrigation amount of 105~120 mm and a nitrogen application amount of 375~400 kg·ha^−1^. This research provides theoretical and technical support for high-yield, high-quality, and efficient irrigation and nitrogen management in solar greenhouses in the North China Plain.

## 1. Introduction

Facility-based vegetable production is a major source of vegetables in Northern China. The rapid development of facility-based vegetable production has reduced the production costs associated with “south-to-north vegetable transportation” in the northern vegetable market [1,2]. Thus, it has gradually became an important component of the rapid agricultural development in this region. The development of facility-based vegetable production has diminished Northern China’s dependence on southern vegetable supplies, thereby circumventing the high transportation costs and postharvest losses inherent in long-distance distribution. Concurrently, it has generated localized employment opportunities for farmers, and, from a long-term perspective, it is poised to enhance agricultural sustainability and elevate the income levels of northern farmers. In recent years, the water resources in the Yellow River Basin have shown a significant and continuous decline, exacerbating issues such as high production inputs, low resource use efficiency, insufficient momentum for increased yields in facility-based vegetable production, and suboptimal agricultural product quality. Moreover, the long-term practice of excessive irrigation and fertilizer application, aimed at maximizing crop yields, has posed significant threats to both soil health and groundwater resources [3,4]. Therefore, the question of how to develop the facility-based vegetable industry under the premise of efficient and limited resource utilization, ensure the effective supply of “vegetable basket” products, and increase local economic incomes has become a critical issue for agricultural production in this region. Cucumber (*Cucumis sativus* L.), as one of the most widely cultivated and popular vegetables in facility-based vegetable production, plays a significant role in addressing this challenge [5]. Cucumber, originating from tropical regions, has a shallow root system primarily concentrated at a soil depth of 10–40 cm. This root architecture facilitates the rapid absorption and utilization of water and nutrients under drip irrigation conditions, while simultaneously rendering the plant highly dependent on and sensitive to optimized water–fertilizer management during production due to its substantial resource demands [6]. Cucumber has significant potential for yield improvement, and increasing the per-mu yield could effectively enhance farmers’ incomes and promote local economic development. The enhancement of its yield and quality primarily depends on rational water and fertilizer management. Currently, most growers, aiming to increase yields and ensure economic benefits, still follow the traditional irrigation and fertilization practice of “plenty of water and frequent fertilizer without consulting others”, resulting in excessive inputs of water, fertilizer, and pesticides in greenhouse production, particularly with nitrogen application far exceeding the crop requirements [7,8,9]. Blindly increasing the irrigation amount and the extent of nitrogen application not only leads to environmental issues such as soil compaction and degradation, nitrate nitrogen accumulation in the soil, and water pollution, but also creates a vicious cycle in greenhouses where improper irrigation and fertilization increase the temperature and humidity—promoting crop diseases, escalating pesticide use, and causing flower and fruit dropping. This ultimately severely affects crop growth, reducing the yields and quality and resulting in low economic efficiency [10,11,12]. Therefore, by investigating the effects of different irrigation and nitrogen application amounts on the cucumber yield and quality and ensuring scientifically rational water and nitrogen usage, it is possible to address the issue of blind irrigation and fertilization in production; this holds significant importance in terms of cost savings, efficiency improvement, and the promotion of sustainable agricultural development.

Water and nitrogen play a decisive role in cucumber production, not only directly participating in the life activities of the plant but also acting as carriers of nutrients, influencing the plant’s absorption and utilization of other substances. In recent years, both domestic and international scholars have conducted extensive research on water and nutrient management for greenhouse cucumbers. Li et al. [13] demonstrated that the combination of water and nitrogen significantly affected the leaf area index and fruit yield of cucumber, with both the leaf area index and yield increasing significantly as the irrigation amount increased. Meanwhile, the leaf area index initially increased and then decreased with higher nitrogen application amounts, and the yield showed a continuous upward trend. Li et al. [14] indicated that the cucumber yield exhibited a positive correlation with the irrigation amount, while the water use efficiency showed a negative correlation with the irrigation amount, meaning that the cucumber yield increased with higher irrigation amounts, whereas the water use efficiency decreased as the irrigation amount increased. The research results of Gong et al. [15] regarding cucumber water consumption showed that pan evaporation ($E_{p-20}$) was highly correlated with cucumber water consumption, and the reference evapotranspiration calculated using the modified Penman–Monteith formula [16] exhibited a highly significant linear relationship with $E_{p-20}$, leading to the recommendation of using 20 cm pan evaporation to guide the drip irrigation schedule for cucumbers in solar greenhouses. Li et al. [17] found that the soluble sugar content of cucumber decreased with increasing irrigation amounts and increased with higher nitrogen application amounts. H. Zhou et al. [18] found that deficit irrigation under certain conditions and rational fertilization significantly improved the fruit quality of economic crops. Wang et al. [19] conducted an optimization assessment of cucumber’s growth and yield based on the environmental temperature, water, and calcium ion application, finding that environmental temperature and water conditions had a more significant impact on cucumber growth. Bello et al. [20] evaluated the irrigation amount and nitrogen application amount for cucumbers with the aim of increasing the yield and reducing nitrogen pollution, finding that deficit irrigation at a 70% nitrogen level was beneficial in improving the cucumber yield in the harsh and arid climate of Qatar. Li et al. [14] optimized water and potassium management for facility-grown cucumbers based on the irrigation amount and potassium application amount, demonstrating that irrigating to 70% of the substrate field capacity’s lower limit and applying 156 mg·L^−1^ of potassium fertilizer facilitated the synergistic improvement of the yield and quality. Most of the above studies evaluated the effects of different irrigation amounts, nitrogen application amounts, or other influencing factors on facility-grown cucumbers, using the yield or quality as a single target. Zhang et al. [21] utilized multiple regression methods to optimize water and fertilizer management, targeting the yield, water use efficiency, and fertilizer partial productivity of spring maize. Zhou [22] employed multiple regression methods to explore the optimal combination of water and nitrogen that maximized the comprehensive benefits in terms of the yield, water use efficiency, and nitrogen agronomic use efficiency for drip-irrigated cotton under mulch in Southern Xinjiang. The current multi-objective optimization research primarily focuses on the synergistic improvement of the yield and water–fertilizer use efficiency, but there is a notable lack of consideration of the fruit quality. In the field of facility agriculture, there is a need to further develop multi-objective optimization strategies for water and nitrogen management in solar greenhouse cucumbers that achieve synergistic improvements in both quantity and quality. Therefore, this study, targeting the typical cultivation patterns of solar greenhouse cucumbers in North China, investigates the effects of different water and nitrogen treatments on the growth, yield, quality, and water–nitrogen use efficiency of facility-grown cucumbers. The goal is to optimize the water and nitrogen management decisions for the synergistic improvement of the yield and quality, as well as ensuring efficient water–nitrogen use efficiency, providing a scientific reference for the water-saving, nitrogen-reducing, high-yield, and high-quality production of greenhouse cucumbers.

## 2. Results

### 2.1. Effects of Water and Nitrogen Treatment on Leaf Area Index of Cucumber

**Figure 1:**
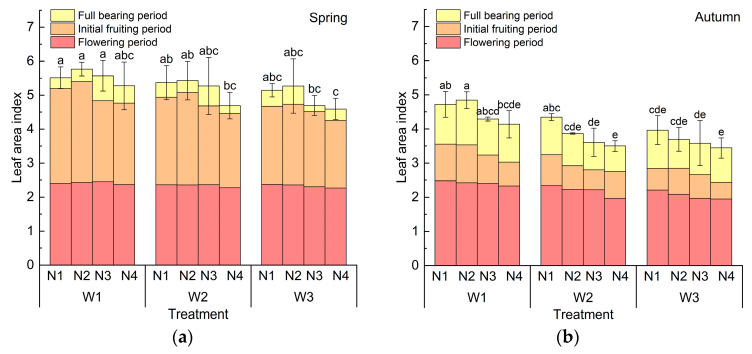
Effects of different water and nitrogen treatments on leaf area index (*LAI*) of cucumber: (**a**) *LAI* in spring; (**b**) *LAI* in autumn. (Note: W1 ($1.0{\;E}_{p-20}$), W2 ($0.75{\;E}_{p-20}$), W3 ($0.5{\;E}_{p-20}$), ${\;E}_{p-20}$ refer to the evaporation measured by a 20 cm evaporation pan; N1: 432 kg·ha^−1^, N2: 360 kg·ha^−1^, N3: 288 kg·ha^−1^, N4: 216 kg·ha^−1^. Different lowercase letters in the figure indicate statistically significant differences among treatments within the same period. The same applies below).

The leaf area index (*LAI*) is an important index reflecting the crop growth status, showing a significant positive correlation with the yield [23,24]. Figure 1 shows the effects of different water and nitrogen treatments on the *LAI* in different growth periods of cucumber. In spring and autumn, the *LAI* showed an increasing trend with the increase in transplanting days, and it finally reached the maximum at the full bearing period. During each growth period, the *LAI* decreased with a decrease in the irrigation amount. The *LAI* changes in spring are shown in Figure 1a, and there was no significant difference among the treatments at the flowering period. At the initial fruiting period, the nitrogen application amount had a very significant effect on the *LAI* (*p* < 0.01). The *LAI* gradually decreased with a decrease in the irrigation amount and showed a trend of first increasing and then decreasing with the decrease in the nitrogen application amount. In all treatments, W1N2 was the largest and W3N4 was the smallest, because, in the initial fruiting period, the plants were sensitive to the water and nutrient requirements in order to ensure the growth of cucumber fruits [25]. In the full bearing period, the influence of the irrigation amount and nitrogen application amount on the *LAI* reached a very significant level (*p* < 0.01). With a decrease in the irrigation amount, the *LAI* gradually decreased, and, compared with W1, the *LAI* of W2 and W3 decreased by 5.69% and 10.35%, respectively. With a decrease in the nitrogen application rate, the *LAI* of cucumber in spring showed a trend of first increasing and then decreasing, among which W1N2 *LAI* was the largest. Meanwhile, W1N1, W1N3, W1N4, W2N1, and W2N2 showed no significant difference compared with W1N2. In conclusion, adequate water and fertilizer amounts are crucial to promote an increase in the *LAI* in spring, but moderate reductions in the irrigation amount and nitrogen application amount have no significant effects on the *LAI*.

The *LAI* of autumn cucumber is shown in Figure 1b. At the flowering period, the irrigation amount had an extremely significant effect on the *LAI* (*p* < 0.001). The *LAI* showed a decreasing trend with the decrease in the irrigation amount. The *LAI* of W1N2 was the largest, followed by W1N1 and W2N1, and the difference between treatments was not significant. At the initial fruiting period, the *LAI* was significantly affected by the irrigation amount (*p* < 0.001) and nitrogen application amount (*p* < 0.01), and it showed a decreasing trend with a decrease in the irrigation amount or nitrogen application amount. At the full bearing period, the *LAI* of autumn cucumber was significantly affected by the irrigation amount (*p* < 0.001) and nitrogen application amount (*p* < 0.01), and it decreased significantly with a decrease in the irrigation amount. Compared with that of W1, the *LAI* of W2 and W3 decreased by 17.87% and 19.86%. W1N2 had the largest *LAI*, followed by W1N1, W2N1, and W1N3. In conclusion, increasing the irrigation amount during the whole growth period of cucumber is beneficial for leaf growth. Increasing nitrogen fertilizer application in the initial fruiting period and full bearing period is beneficial for the growth of cucumber leaves in autumn, but the continued application of nitrogen fertilizer in the full fruit stage would inhibit the growth of cucumber leaves.

### 2.2. Effects of Water and Nitrogen Treatment on Dry Weight of Cucumber

**Figure 2:**
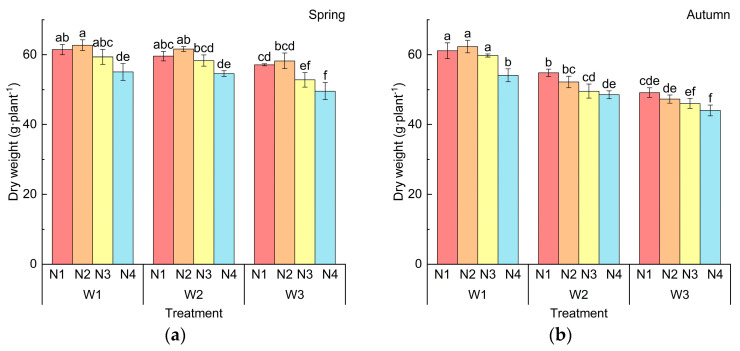
Effects of different water and nitrogen treatments on dry weight (*DW*) of cucumber: (**a**) DW in spring; (**b**) DW in autumn. (Note: W1 ($1.0{\;E}_{p-20}$), W2 ($0.75{\;E}_{p-20}$), W3 ($0.5{\;E}_{p-20}$), ${\;E}_{p-20}$ refer to the evaporation measured by a 20 cm evaporation pan; N1: 432 kg·ha^−1^, N2: 360 kg·ha^−1^, N3: 288 kg·ha^−1^, N4: 216 kg·ha^−1^. Different lowercase letters in the figure indicate statistically significant differences among treatments within the same period).

The crop dry weight (DW) is sensitive to water and fertilizer and is closely related to the crop yield [26]. The variation trend of the DW was consistent with that of the *LAI*. The effects of different water and nitrogen treatments on the DW in spring and autumn are shown in Figure 2. The DW was extremely significantly affected by the irrigation amount (*p* < 0.001), and it decreased with a decrease in the irrigation amount. Compared with W1, the DW of W2 and W3 decreased by 1.09% and 9.21%. In spring, with a constant amount of irrigation, the DW increased first and then decreased with the decrease in nitrogen application. The DW of W1N2 was the largest (62.7 g), followed by W1N1 and W2N2, and the difference among them was not significant. The DW of W3N4 was the smallest, at only 49.58 g. In autumn, the DW showed an obvious decreasing trend with the decrease in the irrigation quantity. Compared with W1, the DW of W2 and W3 decreased by 12.86% and 21.39%. For the W1 treatment, the DW increased first and then decreased with a decrease in the nitrogen application rate. For the W2 and W3 treatments, the DW showed a significant decreasing trend with the decrease in the nitrogen application amount. W1N2 had the highest DW (62.31 g), followed by W1N1 and W1N3, and the differences among them were not significant. In conclusion, for spring cucumber, sufficient water and sufficient fertilizer are beneficial for dry weight accumulation, but excessive nitrogen application would inhibit dry weight accumulation. For autumn cucumber, sufficient water and fertilizer are beneficial for dry weight accumulation, and the influence of the irrigation amount on the dry weight is stronger than that of the nitrogen application amount.

### 2.3. Effects of Water and Nitrogen Treatment on Yield and Yield Components of Cucumber

Table 1 shows the effects of different water and nitrogen treatments on the cucumber yield and yield components. In spring, the cucumber yield and the number of fruit were significantly affected by the irrigation amount, nitrogen application amount, and interaction effect of water and nitrogen, while, in autumn, the cucumber yield and the number of fruit were only significantly affected by the irrigation amount and nitrogen application amount. The yield of cucumbers in spring was significantly higher than that in autumn. Due to the distinct climatic conditions between the two experimental seasons, significant differences were observed in the greenhouse temperature and actual irrigation amounts. Compared with the spring yields, the autumn season showed substantial yield reductions across all nitrogen treatments: 49.38% for N1, 50.67% for N2, 51.28% for N3, and 50.06% for N4. An analysis of the reasons reveals that the spring season features hot weather and abundant sunlight, leading to higher evapotranspiration rates and vigorous photosynthesis in plants, whereas the autumn season is characterized by lower temperatures, fewer sunny days, more overcast days, and insufficient sunlight, resulting in relatively weaker evapotranspiration and photosynthesis in plants. On the 60th day after transplanting, the daily average temperature dropped sharply, failing to meet the light and heat requirements for fruit growth, which led to slow fruit expansion and a decrease in fruit number, ultimately causing the autumn yield to be significantly lower than the spring yield [5,19,27].

In spring, when the irrigation amount was fixed, the yield of cucumbers initially increased and then decreased with the reduction in the nitrogen application amount. When the nitrogen application amount was fixed, the yield of cucumbers showed a significant declining trend as the irrigation amount decreased. Among them, the W1N2 treatment achieved the highest yield of 108.31 t·ha^−1^, followed by W1N1 and W2N2, with minor differences. This phenomenon indicates that high water and high fertilizer levels are beneficial in increasing yields, but moderate water and fertilizer conservation do not significantly reduce the yield of spring cucumbers. In autumn, when the irrigation amount was fixed, the yield of W1 initially increased and then decreased with the reduction in the nitrogen application amount, while the yields of W2 and W3 showed a declining trend as the nitrogen application amount decreased. When the nitrogen application amount was fixed, the autumn yield significantly decreased with the reduction in the irrigation amount. The yields of W2 and W3 decreased by 12.77% and 16.64%, respectively, compared to W1. The W1N2 treatment had the highest yield of 53.81 t·ha^−1^, while the W3N4 treatment had the lowest yield of only 38.80 t·ha^−1^.

When fruits grow abundantly and rapidly, the plant will increase its dependence on water and nutrients [28]. Increasing the irrigation amount not only enhances the soil water potential but also facilitates the absorption of water and nutrients by the plant root system [29]. The differences in the single fruit weight among the treatments were small in both spring and autumn. For spring cucumbers, the number of fruit strips decreased with the reduction in the irrigation amount, and it showed an initial increase followed by a decrease with the reduction in the nitrogen application amount. For autumn cucumbers, in the W1 treatment, the number of fruit strips initially increased and then decreased with the reduction in the nitrogen application amount, while, in the W2 and W3 treatments, it exhibited a declining trend as the nitrogen application amount decreased. The comprehensive results indicate that rational irrigation and nitrogen application contribute to increasing cucumber yields, but excessive nitrogen application will prevent an increase in the cucumber yield.

### 2.4. Effects of Different Water and Nitrogen Treatments on Water and Nitrogen Use Efficiency of Cucumber

The irrigation amount had a highly significant effect on the water consumption of cucumbers in both spring and autumn (*p* < 0.001). Water consumption significantly decreased with a reduction in the irrigation amount, showing a trend of W1 > W2 > W3. In spring, the water consumption of W2 and W3 decreased by 20.37% and 41.89%, respectively, compared to W1. In autumn, the water consumption of W2 and W3 decreased by 24.03% and 49.35% compared to W1. Water consumption initially increased and then decreased with a reduction in the nitrogen application amount. In spring, the W1N4 treatment had the highest water consumption at 284.75 mm, but there was no significant difference compared to W1N3 and W1N2. In autumn, the nitrogen application amount had no significant effect on water consumption across the treatments. The W1N4 treatment had the highest water consumption at 188.05 mm, with no significant differences compared to W1N1, W1N2, and W1N3.

The irrigation amount and nitrogen application amount had a highly significant impact on the water use efficiency (WUE) and partial factor productivity of nitrogen (PFP_n_) of cucumbers in both the spring and autumn seasons (*p* < 0.001). The interaction effect of water and nitrogen had a significant influence on the PFP_n_ in autumn (*p* < 0.05). Under a constant nitrogen application amount, the WUE in both the spring and autumn seasons showed a trend of W3 > W2 > W1. Among these, the W3N1 treatment had the highest WUE in both seasons. In spring, the WUE of the W1 and W2 treatments initially increased and then decreased with a reduction in the nitrogen application amount, while the WUE of the W3 treatment showed a declining trend as the nitrogen application amount decreased. The W3N1 treatment achieved the highest WUE at 49.54 kg·m^−3^. In autumn, the WUE of the W1 treatment initially increased and then decreased with a reduction in the nitrogen application amount, whereas the WUE of the W2 and W3 treatments exhibited a declining trend as the nitrogen application amount decreased. The W3N1 treatment reached the highest WUE at 45.68 kg·m^−3^. The results indicate that, under insufficient irrigation, increasing the nitrogen application amount can enhance the crop’s ability to absorb and utilize water. However, under adequate irrigation, further fertilization may inhibit the crop’s ability to absorb and utilize water. The PFP_n_, an indicator representing the nitrogen use efficiency, significantly increased with a reduction in the nitrogen application amount and significantly decreased with a reduction in the irrigation amount. In both seasons, the highest PFP_n_ was achieved in the W1N4 treatment. In spring, compared to W1, the PFP_n_ of W2 and W3 decreased by 3.69% and 21.52%, respectively. The W1N4 treatment had the highest PFP_n_ at 415.92 kg·kg^−1^, showing no significant difference compared to W2N4. In autumn, compared to W1, the PFP_n_ of W2 and W3 decreased by 13.30% and 17.03%, respectively. The W1N4 treatment had the highest PFP_n_ at 214.23 kg·kg^−1^, demonstrating significant differences compared to the other treatments. In summary, the results indicate that both the water use efficiency and partial factor productivity of nitrogen are significantly influenced by water and nitrogen management. Appropriately reducing the irrigation amount and nitrogen application amount can enhance the water use efficiency, while appropriately reducing the nitrogen application amount can improve the partial factor productivity of nitrogen (Table 2).

### 2.5. Effects of Water and Nitrogen Treatment on Cucumber Quality

#### 2.5.1. Effects of Nitrogen Treatment on Quality-Related Indices of Cucumber

Table 3 presents the results for cucumber-quality-related indicators in response to different water and nitrogen treatments in spring and autumn. Six indicators were measured: total soluble solids (TSS), soluble protein (SP), soluble sugar content (SSC), organic acids (OA), vitamin C (VC), and fruit water content (FW). The results indicate that the irrigation amount had a significant impact on the TSS, SSC, and FW, while the nitrogen application amount significantly affected the OA and FW.

The irrigation amount had a highly significant effect on the TSS of cucumbers in both spring and autumn (*p* < 0.01). The TSS significantly increased with a reduction in the irrigation amount. In spring, compared to W1, the TSS of W2 and W3 increased by 1.69% and 4.96%, respectively. Under a constant irrigation amount, the TSS initially increased and then decreased with a reduction in the nitrogen application amount. The maximum TSS was observed in the W3N2 treatment, while the minimum TSS was recorded in the W1N4 treatment. In autumn, under a constant nitrogen application amount, the TSS of W2 and W3 increased by 1.39% and 3.34%, respectively, compared to W1. As the nitrogen application amount decreased, the TSS initially increased and then decreased. The W3N2 and W3N3 treatments exhibited the lowest TSS, both at 3.40%, while the W1N4 treatment had the lowest TSS at 3.21%. The irrigation amount and nitrogen application amount had a significant impact on the soluble protein (SP) level in spring (*p* < 0.05). The SP increased with higher irrigation amounts and showed an initial rise followed by a decline with increasing nitrogen application. The W1N3 treatment achieved the highest SP content at 1.65 mg·g^−1^, while the W3N1 treatment had the lowest SP content at 1.13 mg·g^−1^. In autumn, the SP content tended to increase with higher irrigation amounts, but the differences among the treatments were not significant (*p* > 0.05). The irrigation amount had a highly significant effect on the SSC (*p* < 0.001). As the irrigation amount decreased, the SSC significantly increased. In spring, compared to W1, the SSC of W2 and W3 increased by 4.07% and 9.76%, respectively. The nitrogen application amount also had a very significant impact on the SSC (*p* < 0.01). As the nitrogen application amount decreased, the SSC significantly decreased. The W3N1 treatment achieved the highest SSC at 13.69 mg·g^−1^, while the W1N4 treatment had the lowest SSC at 11.30 mg·g^−1^. The differences among the treatments were quite significant. In autumn, compared to W1, the SSC of W2 and W3 increased by 9.32% and 12.76%, respectively. As the nitrogen application amount decreased, the SSC initially increased and then decreased. In spring, the OA content was significantly influenced by the nitrogen application amount (*p* < 0.05). The OA initially increased and then decreased with a reduction in the irrigation amount, although the overall trend was not significant. In autumn, the OA content was highly significantly affected by both the irrigation amount and nitrogen application amount (*p* < 0.01). The OA increased with a reduction in the irrigation amount and showed an initial rise followed by a decline with a reduction in the nitrogen application amount. In spring, the differences in the vitamin C (VC) content among the treatments were not significant. In contrast, in autumn, the VC was highly significantly influenced by the nitrogen application amount (*p* < 0.01), showing a decreasing trend as the nitrogen application amount decreased. The fruit weight (FW) was significantly affected by both the irrigation amount and nitrogen application amount in both the spring and autumn seasons (*p* < 0.05). The differences in the FW among the treatments were more pronounced in spring compared to autumn. For any nitrogen application amount, the FW of the W2 treatment was the maximum.

#### 2.5.2. Screening of Representative Indicators of Cucumber Quality

1. Comprehensive evaluation of cucumber quality based on principal component analysis

Due to the multitude of quality indicators, any single quality indicator cannot comprehensively reflect the overall quality of cucumbers. Therefore, it is necessary to conduct a comprehensive analysis and evaluation of various quality indicators of cucumbers. Principal component analysis (PCA) enables this by extracting principal components to transform multiple indicators into a few comprehensive indicators, reducing the dimensionality of the dataset [30]. This method minimizes the loss of original information while achieving the goals of reducing the number of variables and enabling a comprehensive evaluation. PCA is widely applied in comprehensive evaluations within the agricultural field [31]. Due to its clear analytical process and ability to reduce the dataset’s dimensionality, we selected PCA as our analytical approach. However, it should be noted that the PCA results still exhibit some data loss; therefore, they can only provide a reference and directional guidance for optimization strategies. The variables selected for this evaluation are TSS (X_1_), SP (X_2_), SSC (X_3_), OA (X_4_), VC (X_5_), and FW (X_6_), totaling six quality indicators. Principal component analysis (PCA) was employed to perform dimensionality reduction on these six quality indicators through mathematical statistical analysis. The factor loadings and variance contribution rates are presented in Table 4.

In Table 4, the values labeled “X” represent the contributions of each quality indicator to the comprehensive evaluation index. A value closer to “1” indicates that the original quality indicator has a stronger influence on the principal components, with both showing similar trends of variation. A value closer to “−1” indicates that the original quality indicator still has a strong influence on the principal components but exhibits opposite trends of variation. For the spring data, X3 has the strongest influence on the primary principal component (PC1) and shares a similar trend of variation. The variance contribution rates indicate the degree to which the comprehensive evaluation index influences the total evaluation, with larger values representing a greater impact. The cumulative contribution rate is the cumulative sum of the variance contribution rates. The number of observations is the same for all analyzed variables to avoid statistical errors.

In spring, the eigenvalues of the primary and secondary principal components were 1.67 and 1.30, respectively. However, their cumulative contribution rate was only 74.7%. According to scholars such as Wang et al. [32], the cumulative variance contribution rate of the principal components must meet the requirement of >85%. Therefore, it was necessary to include a third principal component. The final cumulative variance contribution rate reached 90.47%, allowing the first three principal components to replace the six quality indicators for the evaluation of cucumber quality. The primary principal component explained 46.56% of the total original information, mainly reflecting the influence of four indices: TSS (X1), SP (X2), SSC (X3), and VC (X5). The secondary principal component contained 28.13% of the original information, which mainly explained the influence of the TSS (X1), SP (X2), and OA (X4). The tertiary principal component explained 15.77% of the original information, mainly influenced by the OA (X4).

In autumn, the variance contribution rate of the first three principal components reached 87.53%, so they were used instead of the six quality indices to evaluate the quality of cucumbers. The primary principal component explained 48.48% of the original information, which mainly reflected the influence of four indicators: TSS (X1), SP (X2), SSC (X3), and OA (X4). The secondary principal component explained 24.29% of the original information and was mainly influenced by VC (X5). The tertiary principal component explained 14.75% of the original information and was mainly influenced by FW (X6).

According to examples 6 and7, the formula for the calculation of the principal component score can be derived from the data in Table 4. After data calculation, the comprehensive scores are as shown in Table 5. The results indicate that the comprehensive quality scores followed the trend of W3 > W2 > W1, demonstrating that low-water treatments (W3) produced cucumbers with the highest quality. This suggests that increased irrigation amounts negatively impact cucumber quality, revealing a negative correlation between the irrigation amount and fruit quality. This phenomenon may be attributed to excessive irrigation hindering the accumulation of key compounds in fruits.

The results in Table 4 demonstrate that, in spring, the TSS had the greatest influence on the comprehensive evaluation in the principal component analysis, followed by the SSC. In autumn, the SSC exhibited the strongest impact. The differences between the SSC and TSS were relatively minor, and the correlations among the indicators remain unclear, necessitating further in-depth analysis.

2. Screening of representative quality indices of cucumber

The PCA analysis results (Table 4) indicate that TSS and SSC exert the most significant influence on the comprehensive assessment in PCA, suggesting their strong potential as representative indicators for quality evaluation. To select appropriate indicators for the comprehensive evaluation of water and nitrogen management, a correlation analysis was conducted among the six quality indicators: TSS, SSC, VC, SP, SA, and FW. The correlation coefficients and their significance levels are presented in Table 6. The values in Table 6 indicate that the closer the number is to 1, the stronger the positive correlation between the two indicators; conversely, the closer it is to −1, the more pronounced the negative correlation [33]. In spring, both TSS and SSC show weak negative correlations with FW but weak positive correlations with SA and VC. A highly significant (*p* < 0.001) positive correlation is observed between TSS and SSC. While TSS exhibits a weak negative correlation with SP, SSC demonstrates a significant (*p* < 0.05) negative correlation with SP. In autumn, both TSS and SSC showed very weak negative correlations with VC, while exhibiting weak positive correlations with FW. A statistically significant positive correlation (*p* < 0.05) was observed between TSS and SSC. TSS demonstrated a positive correlation with SP but a significant negative correlation (*p* < 0.05) with SA. SSC showed a significant negative correlation (*p* < 0.05) with SP and a non-significant negative correlation with SA. The summation of the absolute values of the correlation coefficients was conducted, and the results are presented in Table 6. For both the spring and autumn seasons, the cumulative absolute values of SSC surpassed those of TSS. Therefore, SSC demonstrated the strongest correlation with the other quality indicators.

A comprehensive evaluation integrating the PCA results (Table 4) and inter-indicator correlation analyses (Table 6) demonstrated that SSC is the most suitable parameter to serve as a composite quality indicator.

### 2.6. Optimization of Production Management Scheme Based on Yield, Water Use Efficiency, Partial Factor Productivity of Nitrogen, and Quality

Bivariate quadratic regression models were established with the cucumber yield, water use efficiency (WUE), partial factor productivity of nitrogen (PFP_n_), and soluble sugar content (SSC) as dependent variables and the nitrogen application amount and irrigation amount as independent variables for both the spring and autumn seasons. The regression equations, degree of fitting (R^2^), and corresponding optimal water–nitrogen treatments for the maximum values are present in Table 7.

In spring, the maximum cucumber yield was achieved at a 285.03 mm irrigation amount and a 401.14 kg·ha^−1^ nitrogen application amount. The maximum WUE occurred at a 176.15 mm irrigation amount and a 405.55 kg·ha^−1^ nitrogen application amount. The maximum PFP_n_ was obtained at a 278.23 mm irrigation amount and a 216.00 kg·ha^−1^ nitrogen application amount. The maximum SSC was observed at a 176.15 mm irrigation amount and a 423.00 kg·ha^−1^ nitrogen application amount.

In autumn, the maximum yield was attained at a 189.50 mm irrigation amount and a 414.37 kg·ha^−1^k nitrogen application amount. The maximum WUE occurred at a 94.75 mm irrigation amount and a 432.00 kg·ha^−1^ nitrogen application amount. The maximum PFP_n_ was achieved at a 189.50 mm irrigation amount and a 216.00 kg·ha^−1^ nitrogen application amount. The maximum SSC was recorded at a 96.68 mm irrigation amount and a 317.39 kg·ha^−1^ nitrogen application amount.

These results indicate that, under identical water and nitrogen management conditions, it is impossible to simultaneously maximize the yield, WUE, PFP_n_, and SSC (Table 7). Therefore, it is not feasible to determine the optimal water and nitrogen management strategy directly based on the equations, necessitating further analysis.

Further analysis was performed using spatial analysis to evaluate the confidence intervals (95%, 90%, 85%, 80%, 75%, and 70%) of the maximum values for the yield, water use efficiency (WUE), partial factor productivity of nitrogen (PFP_n_), and soluble sugar content (SSC). Contour maps were generated to visually assess these intervals. As the confidence intervals expanded, the overlapping shaded regions for the yield, WUE, and SSC gradually increased. However, PFP_n_ exhibited an inverse trend compared to the yield, WUE, and SSC, resulting in no overlap between the 95%, 90%, 85%, and 80% confidence intervals of the PFP_n_ maxima and those of the yield and WUE. Extending the optimization range further would significantly reduce the yield and WUE, which contradicts the optimization objectives of high yields and water conservation. Therefore, only the yield, WUE, and SSC were considered in the optimization analysis [21].

**Figure 3:**
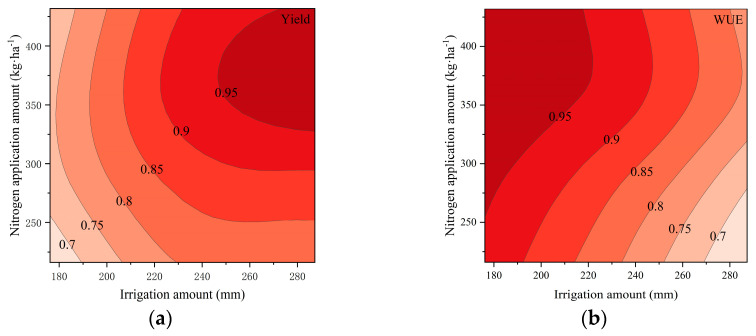

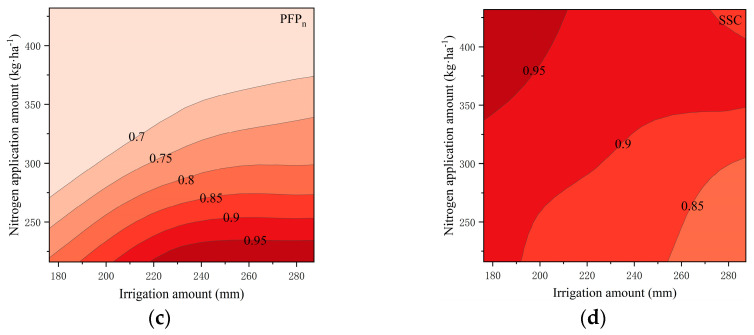
Relationship between irrigation amount and nitrogen application amount and relative yield, relative WUE, relative PFP_n_, and relative SSC in spring: (**a**) relative yield; (**b**) relative WUE; (**c**) relative PFP_n_; (**d**) relative SSC. (Note: 0.95, 0.9, 0.85, and 0.8 denote the ratios of each index to its maximum value. The relative yield, relative WUE, relative PFP_n_, and relative SSC are values between 0 and 1 after normalizing for various indices).

**Figure 4:**
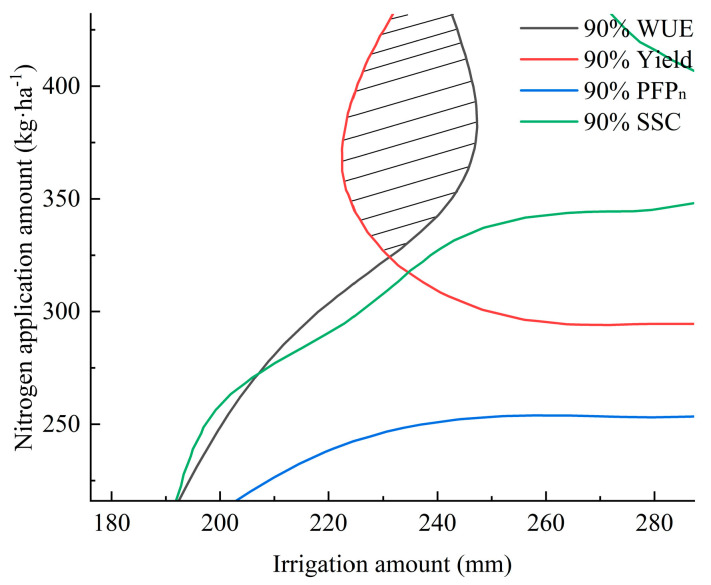
Comprehensive evaluation of yield, WUE, PFP_n_, and SSC (spring). (Note: The shaded area in the figure represents the acceptable region for evaluation).

For spring cucumber cultivation, overlapping regions were observed within the 90% confidence intervals of the yield and WUE, which also intersected with the 90% confidence interval of the SSC (Figure 3). This intersection zone satisfied the optimization criteria. The 90% contour lines of the yield, WUE, PFP_n_, and SSC were extracted and plotted (Figure 4), with their intersection representing the optimal range. To align with the objectives of water saving and fertilizer reduction, the recommended irrigation and nitrogen application amounts were 225~240 mm and 350~380 kg·ha^−1^, respectively.

**Figure 5:**
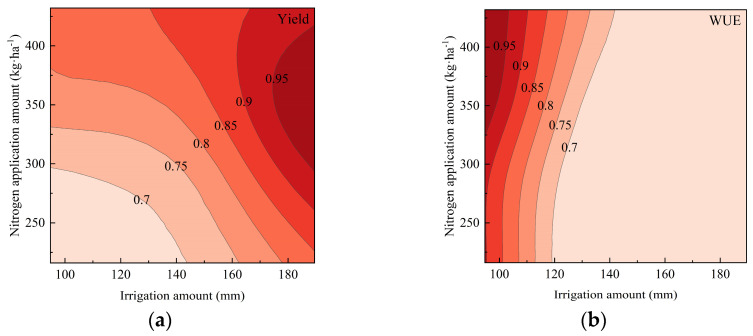

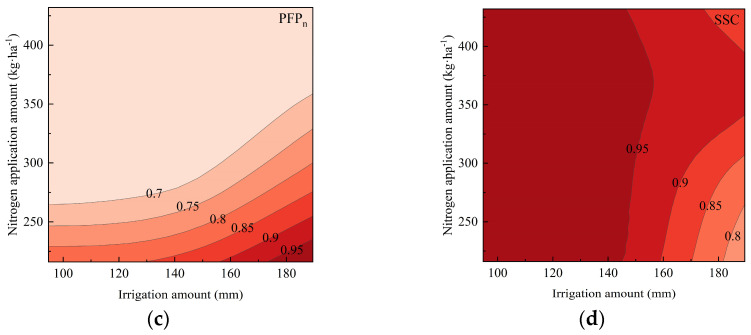
Relationship between irrigation amount and nitrogen application amount and relative yield, relative WUE, relative PFP_n_, and relative SSC in autumn: (**a**) relative yield; (**b**) relative WUE; (**c**) relative PFP_n_; (**d**) relative SSC.

**Figure 6:**
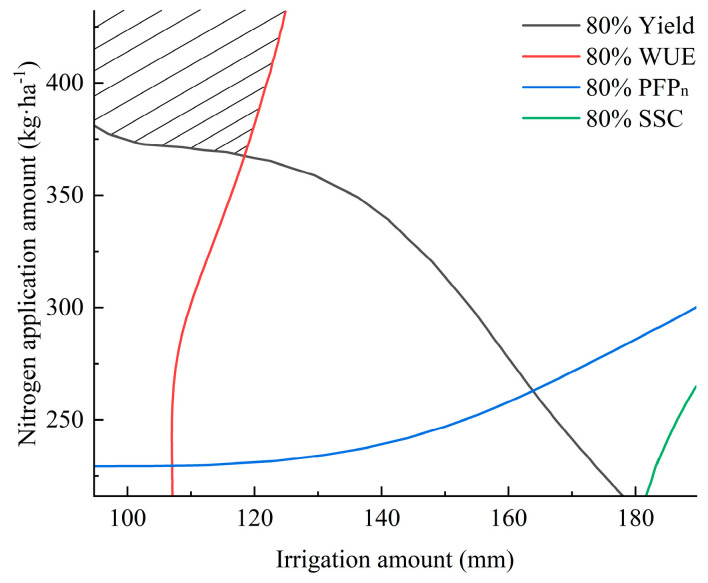
Comprehensive evaluation of yield, WUE, PFP_n_, and SSC (autumn). (Note: The shaded area in the figure represents the acceptable region for evaluation).

For autumn cucumber cultivation, schematic diagrams of the 80% confidence interval contours for the yield, WUE, and SSC are illustrated in Figure 5. As is shown, the yield and WUE exhibited overlapping regions within their 80% confidence intervals. The 80% contour lines of all four parameters (yield, WUE, PFP_n_, and SSC) were further plotted (Figure 6). Due to the extensive overlap area, the optimal water–nitrogen combinations were selected based on the conservation priorities: irrigation amounts of 105~120 mm and nitrogen application amounts of 375~400 kg·ha^−1^.

## 3. Discussion

Soil moisture is the foundation for soil nutrient utilization, and fertilization can improve the crop water use efficiency. An appropriate combination of water and nitrogen not only helps to control resource inputs, protect land resources, and preserve the ecological environment, but also improves the fruit quality and enhances the water and nitrogen use efficiency while ensuring the crop yield, thereby achieving high-quality agricultural production [21].

This study reveals that different water and nitrogen treatments have varying degrees of impact on the leaf area index (*LAI*), dry weight (DW), and yield of cucumbers. As the irrigation amount increases, the *LAI*, DW, and yield of cucumbers show an upward trend, expressed as W1 > W2 > W3. This indicates that, under the same nitrogen treatment, increasing the irrigation amount promotes plant growth and fruit maturation. This is because increasing the irrigation amount enhances the soil water potential, facilitating the absorption and utilization of water by the roots [29]. The water and nitrogen treatment experiments performed in this study showed that, under high-water conditions, the *LAI* initially increased and then decreased with a reduction in the nitrogen application amount—a trend that is consistent with the findings of Li et al. [13]. This may be because an excessive nutrient supply can cause salt stress, inhibiting nutrient absorption, while an insufficient nutrient supply fails to meet the growth demands of the plant, hindering nutrient absorption and accumulation [34]. In this experiment, under different water and nitrogen treatments, the dry weight initially increased and then decreased with an increasing nitrogen application amount—a trend that is inconsistent with the findings of Li et al. [13], who reported that the dry matter increased with both the irrigation amount and nitrogen application amount. This discrepancy may be related to differences in the cucumber varieties and external meteorological conditions in the planting regions. Wang et al. [35] found that increasing both the irrigation amount and nitrogen application amount promoted the cucumber yield, with the maximum yield achieved under high-water and high-nitrogen treatments. According to the research of Bello et al. [20], when the nitrogen application amount is excessively high, the yield significantly decreases. The results of this experiment indicated that the cucumber yield significantly increased with higher irrigation amounts, but, in the high-water treatments for both spring and autumn cucumbers, the yield initially increased and then decreased with increasing nitrogen application amounts—a trend that differs from the findings mentioned above. The reason for this phenomenon may be that excessive nitrogen application leads to the accumulation of NO_3_^−^-N in the root zone, limiting the crop’s ability to absorb and utilize nutrients and water [36]. Only by maintaining the nitrogen application amount and irrigation amount within an appropriate range can a high yield be sustained while reducing resource inputs [37,38].

Different water and nitrogen treatments have varying degrees of impact on the water and nitrogen use efficiency of cucumbers. Generally, the water use efficiency (WUE) decreases with increasing irrigation amounts, and, when the irrigation amount is coordinated with the yield, it can effectively improve the crop WUE. Zhang et al. [39] suggested that, under greenhouse drip irrigation conditions, $0.8{\;E}_{p-20}$ can achieve higher WUE. Zhao et al. [40] found that the effect of the interaction between the irrigation amount and fertilization amount on the WUE is antagonistic, meaning that increasing irrigation amounts reduce the WUE, while increasing fertilization amounts improve the WUE. In the present experiment, the water use efficiency (WUE) in high- and medium-water treatments for spring cucumbers and high-water treatments for autumn cucumbers initially increased and then decreased with a reduction in the nitrogen application amount. This may be because excessive nitrogen application leads to an increase in the nitrogen concentration in the root zone, hindering the plant roots’ ability to absorb and utilize water [36]. The partial factor productivity of nitrogen (PFP_n_) is significantly influenced by the irrigation amount and nitrogen application amount but is almost unaffected by the interaction effect of water and nitrogen, which is consistent with the findings of Li et al. [41]. Under different water and nitrogen treatments, the PFP_n_ decreases with a reduction in the irrigation amount. This is because irrigation helps to enhance the absorption and utilization of nutrients by cucumbers and promotes the transformation and utilization of nitrogen fertilizers [42]. It may also be because, under the same nutrient conditions, the yield significantly increases with higher irrigation amounts. Excessive nitrogen application significantly reduces the PFP_n_, likely because it exceeds the nitrogen demand of cucumber plants and is accompanied by nitrogen leaching and volatilization, ultimately leading to a decrease in the cucumber yield [43].

The effects of water and nitrogen treatments on cucumber quality vary. The irrigation amount significantly impacts the total soluble solids and soluble sugar content, both of which show a decreasing trend with increasing irrigation amounts, consistent with the findings of Gong et al. [15] and Wu et al. [38]. Numerous studies have demonstrated that water deficit conditions enhance fruit quality, although the yield may decrease, due to the crop’s improved ability to synthesize fruit-related compounds, thereby increasing the total soluble solids and soluble sugar content, whereas an increased water supply may dilute the compounds within the fruit [44,45], leading to a decline in fruit quality. In this experiment, principal component analysis (PCA) was used to comprehensively evaluate the fruit quality of cucumbers. Six quality indicators were selected as evaluation variables: the total soluble solids (TSS), soluble protein content (SP), soluble sugar content (SSC), organic acid content (OA), vitamin C content (VC), and fruit water content (FW). Based on the final comprehensive scoring results (Table 5), the ranking was W3 > W2 > W1, indicating that low-water treatments are beneficial in improving cucumber quality. According to the results (Table 4), the TSS and SSC contributed the most to the three principal components, and the SSC showed stronger correlations with the other five quality variables. Therefore, the SSC was selected as the cucumber quality indicator for the optimization of the management strategy.

Using the irrigation amount and nitrogen application amount as independent variables, binary quadratic surface fitting was performed for the yield, SSC, WUE, and PFP_n_ of cucumbers in both seasons. Spatial analysis was then applied to analyze and optimize the water and nitrogen management strategies for cucumbers in both seasons. Further analysis revealed the recommended water and nitrogen management combination for spring cucumbers was an irrigation amount of 225~240 mm and a nitrogen application amount of 350~380 kg·ha^−1^; for autumn cucumbers, the recommended combination is an irrigation amount of 105~120 mm and a nitrogen application amount of 375~400 kg·ha^−1^. The recommended water and nitrogen management combinations can ensure the cucumber yield while maintaining its quality and water use efficiency, achieving the optimization goal of a synergistic improvement in both quantity and quality.

Unfortunately, this study was conducted only during the spring and autumn growing seasons of 2024, with a short experimental duration and insufficient replication. Consequently, the findings lack statistical representativeness and broader generalizability. If more statistically significant representative conclusions need to be obtained, long-term experimental work spanning multiple growing years is required to account for interannual variability and validate the reproducibility. The planting seasons for cucumber crops differ, with varying meteorological conditions and significant differences in the actual irrigation amounts and sunlight availability, leading to different optimization results. To further achieve the goal of a synergistic improvement in quantity and quality, longer-term experimental validation is required.

## 4. Materials and Methods

### 4.1. Experimental Site

The experiment was conducted from April to November 2024 for both spring and autumn cucumber crops in the solar greenhouse at the Xinxiang Comprehensive Experimental Base of the Chinese Academy of Agricultural Sciences (35°9′ N, 113°47′ E, altitude 78.7 m) in Qiliying Town, Xinxiang City, Henan Province. The greenhouse measured 60 m in length and 8.5 m in width, with the soil surface located 0.5 m above ground level, with an east–west orientation, and the crop rows were in the north–south direction. The site had a warm temperate continental monsoon climate with a long-term annual mean air temperature of 14.1 °C. The mean annual precipitation was 548.3 mm, while the mean annual evaporation was 1908.7 mm (value of 20 cm evaporation pan). The frost-free period was 200.5 d, with a mean annual sunshine duration of 2398.8 h. The greenhouse was covered with a polyethylene film. The wall of the greenhouse was inlaid with 0.6 m thermal insulation materials, and there were no other heating facilities in the greenhouse. The soil texture was silty loam. The average bulk density of the soil at a 0–60 cm depth was 1.59 g·cm^−3^, the pH was 8.5, the organic matter content was 0.85%, the total alkali-hydrolyzable nitrogen was 48.34 mg·kg^−1^, the available phosphorus was 29.32 mg·kg^−1^, and the available potassium was 257.8 mg·kg^−1^. The field water holding rate was 22.97% (mass fraction).

### 4.2. Experimental Design

**Figure 7:**
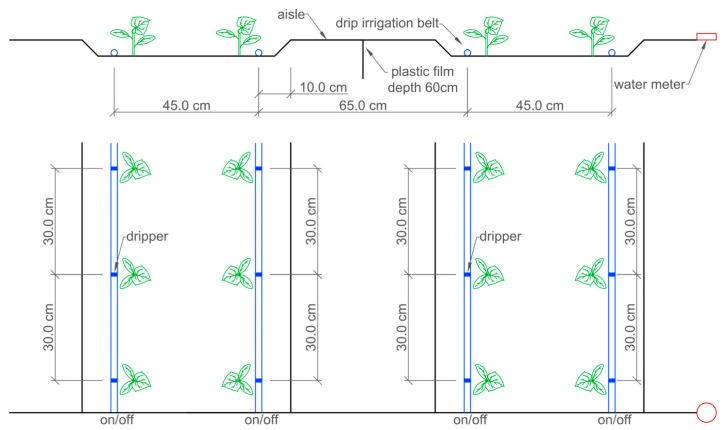
Schematic illustration of the experimental plots and the drip irrigation system. Note: The black lines represent the soil and impermeable plastic film, the blue line represents the plastic drip irrigation strips, the blue rectangular area represents the drip head position, the red line represents the water meter, and the green images represent plants. The data annotations are as illustrated in the figure.

Cucumber (*Cucumis sativus* L.) was selected as the experimental crop, and the cucumber variety was “Ruyi”. The plantiPng pattern adopted a wide–narrow row arrangement, with wide rows of 65 cm, narrow rows of 45 cm, and plant spacing of 30 cm, resulting in a planting density of 5.7 plants·m^−2^. This study adopted an alternating wide–narrow row planting pattern for cucumber cultivation. Two rows of cucumbers were planted in the narrow rows, with row spacing of 45 cm. Drip irrigation tapes were laid along the cucumber rows in the narrow rows, with the emitter spacing set at 30 cm to match the plant spacing of the cucumbers, thereby facilitating more precise irrigation and fertilization. The wide rows served as operational channels for manual harvesting, pest control, and data collection. The implementation of this wide–narrow row arrangement not only enhanced the ventilation between the plants but also improved the light utilization for the middle and lower canopies of the plants. Cucumber seedlings were transplanted when they reached the three-leaf/one-heart stage. The spring crop experiment began on 10 April 2024 and ended on 3 July, while the autumn crop experiment started on August 18 and concluded on 8 November. The experiment was designed with two factors: the irrigation amount and nitrogen application amount. The irrigation amounts were referred to as the $E_{p-20}$, measured by a 20 cm pan (0.20 m in diameter and 0.11 m in depth), with three levels set as follows: W1 ($1.0E_{p-20}$), W2 ($0.75E_{p-20}$), and W3 ($0.5E_{p-20}$). The pan was placed 10 cm above the plant canopy, and evaporation was measured daily at 8:00 a.m. using a glass-graduated flask. Then, the 20 cm pan was cleaned and refilled with 20 mm fresh water using a glass-graduated flask. When the $E_{p-20}$ reached 20 ± 2 mm, irrigation was applied for all treatments. This decision was supported by our team’s previous research on irrigation scheduling for tomato crops in solar greenhouses. Liu et al. [46] found that initiating irrigation when the cumulative evaporation ($E_{p-20}$) reached 20 mm represented an optimal frequency that not only promoted high yields but also enhanced the water use efficiency in greenhouse production systems. Although this evaporation pan is not a standard pan used worldwide, it is recommended in China to measure evaporation as a reference [46,47,48]. The nitrogen application amount was set at four levels: N1 (432 kg·ha^−1^), N2 (360kg·ha^−1^), N3 (288 kg·ha^−1^), and N4 (216 kg·ha^−1^). The experiment followed a completely randomized block design with 12 treatments, each replicated three times. Each plot measured 8 m in length and 2.2 m in width. Drip irrigation combined with integrated water and fertilizer management was employed, with drip lines installed along the cucumber rows. Intra-strip emitters were spaced at 30 cm intervals, delivering a flow rate of 1.38 L·h^−1^ per emitter. Each plot was equipped with a high-precision water meter (accuracy: 0.001 m^3^) to ensure the accurate control of the irrigation volume. To prevent lateral water seepage from interfering with the experimental results, plastic film barriers were buried to a depth of 60 cm between adjacent plots (Figure 7).

The total irrigation amounts for both seasons are shown in Table 8. In the spring season, the irrigation amounts for each treatment were W1: 287.3 mm, W2: 231.73 mm, and W3: 176.15 mm. In the autumn season, the irrigation amounts were W1: 189.5 mm, W2: 142.13 mm, and W3: 94.75 mm. The fertilizer application rates are shown in Table 9. During land preparation, all treatments received a basal application of organic fertilizer at 9.1 t·ha^−1^, nitrogen and potassium fertilizers with a basal-to-topdressing ratio of 3:7 in spring and 2:8 in autumn, and phosphorus fertilizer (P_2_O_5_) at 120 kg·ha^−1^. The basal fertilizers were applied uniformly by broadcasting. Urea (N: 46.4%) was used as the nitrogen fertilizer, superphosphate (P_2_O_5_: 16%) as the phosphorus fertilizer, and potassium sulfate (K_2_O: 52%) as the potassium fertilizer. In the spring season, a total of 14 irrigations were applied, with the topdressing of the nitrogen fertilizer starting at the flowering stage and applied five times through drip irrigation. In the autumn season, a total of 10 irrigations were applied, with the topdressing of the nitrogen fertilizer starting at the flowering stage and applied five times through drip irrigation. To ensure the survival of the seedlings after transplantation, all treatments were irrigated once immediately.

### 4.3. Test Observation Items and Methods

#### 4.3.1. Greenhouse Meteorological Factors

**Figure 8:**
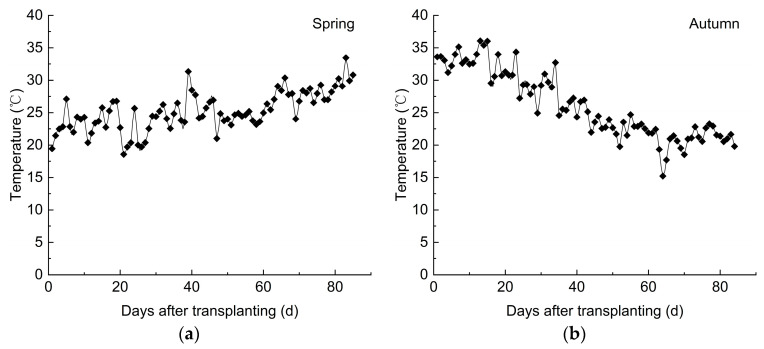
The variation trend of the daily average temperature inside the greenhouse. (**a**) Daily average temperature in spring; (**b**) daily average temperature in autumn.

An automatic weather monitoring system (CR1000, Campbell, CA, USA) was installed both inside and outside the greenhouse to measure meteorological parameters such as the air temperature (Ta), relative humidity (RH), and total solar radiation (Rs). Data were collected at 30 min intervals. The daily average temperature inside the greenhouse during the experimental period is shown in Figure 8. In spring, the daily average temperature gradually increased with the number of days after transplanting, rising from 20 °C to 30 °C, with the highest daily average temperature reaching approximately 32.5 °C. In autumn, the daily average temperature gradually decreased with the number of days after transplanting, fluctuating from 36 °C down to 15 °C. There were approximately 7 d when the daily average temperature fell below the optimal growth temperature of 20 °C [49].

#### 4.3.2. Soil Moisture and Computational Methods

At the end of the experimental period, the soil moisture at depths of 0–60 cm was measured in different plots. The crop evapotranspiration (ET, mm) was calculated using the following water balance equation [41]:

(1) ET=P+I+U−D−R−∆W

where P is the precipitation (mm), I is the irrigation amount (mm), U is the water movement from the deep soil into the root zone (mm), D is the deep percolation (mm), R is the surface runoff (mm), ∆W is the change in soil water storage within the 0–60 cm soli layer (mm), and ∆W is calculated as follows:

(2) $$∆W=1000\times h(Q_{t2}-Q_{t1})$$

where h is the crop root depth (m), and $Q_{t1}$ and $Q_{t2}$ are the average soil water content of the root zone at t1 and t2, respectively (cm^3^·cm^−3^).

The experiment was conducted in a greenhouse, so P=0, D=0, and R=0. The groundwater table was below 5.0 m, so the crop roots could not absorb and utilize the groundwater. Thus, the underground water recharge was negligible (U=0). In summary, example 1 could be simplified as

(3) ET=I−∆W

#### 4.3.3. Leaf Area Index

After entering the flowering period, nine representative cucumber plants free from pests and diseases were selected from each treatment. A ruler with accuracy of 0.1 cm was used to measure the leaf length and maximum leaf width of each leaf on the selected plants. The leaf area of the cucumbers was calculated using an empirical formula, with a correction coefficient of K = 0.743 [50], to estimate the leaf area index (*LAI*) of the entire plot. Measurements were taken once during the flowering period, the initial fruiting period, and the full bearing period, respectively.

#### 4.3.4. Aboveground Dry Weight

At the end of the experiment, three healthy cucumber plants were selected from each treatment. The plants were separated at the stem base from the underground parts, cleaned of dirt, and divided into organs. They were then placed in an oven at 105 °C for 0.5 h to deactivate enzymes, followed by drying at 75 °C until a constant weight was achieved. The total aboveground dry matter mass per plant was measured by an electronic balance with accuracy of 0.01 g.

#### 4.3.5. Yield and Fruit Quality

Cucumbers were harvested after entering the initial fruiting period, with the yield measured every 2~3 days. In each replication, 20 consecutive cucumber plants with uniform growth and free from diseases and pests were selected for yield measurement. Fruits were harvested and recorded when their length reached 30~40 cm, and they were weighed with an electronic balance with accuracy of 5 g. Each treatment was repeated three times.

For quality assessment, after entering the peak fruiting period, three cucumber fruits with consistent maturity were selected for measurement approximately every 14 days following harvest. Each treatment was repeated three times. We homogenized the fruit using a blender and separately measured the total soluble solids (TSS), vitamin C (VC), soluble protein (SP), soluble sugar content (SSC), and organic acids (OA).

TSS was measured using a handheld refractometer (PR-32 α, ATAGO, Tokyo, Japan) [41]. VC was measured using the molybdenum blue colorimetric method [51]. SP was measured using the Coomassie brilliant blue method [52]. SSC was measured using the anthrone colorimetric method [46]. OA was measured by titration with 0.1 mol·L^−1^ NaOH [53].

### 4.4. Water and Nitrogen Use Efficiency of Crops

The formula for water use efficiency (WUE) is described as follows [54]:

(4) $$WUE=\frac{Y_{a}}{ET}\times 100$$

where WUE is the water use efficiency (kg·m^−3^); $Y_{a}$ is the total fruit economical yield (t·ha^−q^); and ET is the crop evapotranspiration (mm).

The partial factor productivity of nitrogen (${PFP}_{n}$, kg·kg^−1^) is one of the indicators of nitrogen use efficiency and was calculated as follows [51]:

(5) $${PFP}_{n}=\frac{Y_{a}}{NFR}\times 1000$$

where NFR is the nitrogen application amount (t·ha^−1^).

### 4.5. Methods for Evaluation of Cucumber Comprehensive Fruit Quality

Principal component analysis (PCA) was used to evaluate the comprehensive fruit quality. The calculation formulas for the scores of each principal component and the comprehensive score are shown below:

(6) $$F_{i}=U_{1i}X_{1}+U_{2i}X_{2}+\cdots +U_{pi}X_{p}$$

(7) $$F=W_{1}F_{1}+W_{2}F_{2}+\cdots +W_{i}F_{i}$$

where $F_{i}$ is the score of the i-th principal component; $U_{1i}$, $U_{2i}$, ⋯, $U_{pi}$ are the score coefficients of the *i*-th principal component; $X_{p}$ is the standardized value; F is the comprehensive score of the principal components; and $W_{i}$ is the weight of the *i*-th principal component, which corresponds to the contribution rate of each principal component factor.

### 4.6. Data Analysis

Excel 2019 was used for data processing, Origin 2022 (OriginLab Inc., Hampden, MA, USA) was used for graphic design, and the R Programming Language software was used for the analysis of variance (ANOVA) and the calculation of the contribution rates and cumulative contribution rates of the principal components (PCA). A comparison of the treatment means was carried out using Duncan ’s multiple-range test at a 5% probability level. Different lowercase letters in the graphs and tables indicate significant differences (*p* < 0.05).

## 5. Conclusions

In this study, under different water and nitrogen treatments with integrated drip irrigation and fertilization, facility-grown cucumbers were used as the research subjects. The low-water treatment significantly improved the cucumber fruit quality and crop water use efficiency, but reduced the leaf area index, dry matter accumulation, and yield. Excessive nitrogen application amounts (above 360 kg·ha^−1^) may adversely affect the fruit yield and leaf area index development, while showing minimal impacts on fruit quality. A comprehensive analysis using principal component analysis and correlation coefficient methods revealed that the soluble sugar content (SSC) served as the most suitable indicator for the evaluation of the overall cucumber quality. For cucumbers cultivated in solar greenhouses across the North China Plain, the optimal water and nitrogen application amounts are 225~240 mm and 350~380 kg·ha^−1^ during the spring season, respectively, while 105~120 mm and 375~400 kg·ha^−1^ are recommended for autumn cultivation.

However, as these conclusions were derived from a single-year experiment, the findings may lack generalizability and representativeness. Further multi-year studies are required to validate the results presented in this manuscript.

## Figures and Tables

**Table 1:** Effects of different water and nitrogen treatments on cucumber yield and yield components in spring and autumn.

Season	Treatment		Yield (t·ha^−1^)	Single Fruit Weight (g)	Number of Fruit Strips
Spring	W1	N1	105.07 ab	198.31 abc	9.75 a
		N2	108.31 a	204.12 ab	9.88 a
		N3	97.95 cd	207.53 a	8.66 bcd
		N4	89.84 e	205.85 ab	8 fg
	W2	N1	98.99 cd	204.05 ab	8.9 bc
		N2	101.63 bc	205.99 ab	9.05 b
		N3	95.37 d	204.68 ab	8.54 cde
		N4	88.47 e	200.54 abc	8.09 efg
	W3	N1	78.21 f	205.69 ab	7.61 g
		N2	81.33 f	197.96 abc	8.22 def
		N3	79.97 f	196.22 bc	7.8 fg
		N4	72.53 g	193.21 c	7.01 h
	Significant level (F value)				
	W		317.812 ***	134.27 *	85.151 ***
	N		56.821 ***	23.16 ns	41.754 ***
	W × N		4.107 **	2.074 ns	4.455 **
Autumn	W1	N1	50.41 bc	177.65 b	5.20 a
		N2	53.81 a	181.22 ab	5.44 ab
		N3	50.8 b	183.56 ab	5.07 b
		N4	46.27 de	188.05 a	4.51 cd
	W2	N1	47.55 cd	184.96 ab	4.71 c
		N2	45.69 de	183.26 ab	4.70 c
		N3	42.15 fg	177.30 b	4.33 de
		N4	40.2 gh	175.71 b	4.03 ef
	W3	N1	44.91 def	180.44 ab	4.56 cd
		N2	44.16 e	182.78 ab	4.36 de
		N3	39.93 gh	182.99 ab	4.00 f
		N4	38.8 h	178.40 ab	3.98 f
	Significant level (F value)				
	W		78.902 ***	0.350 ns	69.005 ***
	N		26.342 ***	0.100 ns	27.988 ***
	W × N		2.203 ns	52.88 *	1.908 ns

Note: Different lowercase letters in the same column indicate significant differences between different treatments, * indicates a significance level of *p* < 0.05, ** indicates a significance level of *p* < 0.01, *** indicates a significance level of *p* < 0.001, and ns indicates no significant effect (*p* > 0.05). The same applies below.

**Table 2:** Effects of water and nitrogen treatments on water consumption, WUE, and PFP_n_ in spring and autumn.

Season	Treatment		Water Consumption (mm)	WUE (kg·m^−3^)	PFP_n_ (kg·kg^−1^)
Spring	W1	N1	276.49 b	38.01 d	243.22 e
		N2	280.49 ab	38.62 d	300.86 c
		N3	282.61 ab	34.67 e	340.09 b
		N4	284.75 a	31.59 f	415.92 a
	W2	N1	219.69 c	45.09 b	229.14 f
		N2	223.73 c	45.44 b	282.31 d
		N3	227.26 c	41.97 c	331.14 b
		N4	224.62 c	39.39 cd	409.57 a
	W3	N1	157.91 e	49.54 a	181.05 g
		N2	166.62 d	48.84 a	225.91 f
		N3	169.93 d	47.07 ab	277.66 d
		N4	158.91 e	45.67 b	335.78 b
	Significant level (F value)				
	W		2107.540 ***	177.435 ***	242.80 ***
	N		5.755 **	24.498 ***	672.90 ***
	W × N		1.432 ns	0.854 ns	1.7 ns
Autumn	W1	N1	186.82 a	26.99 gh	116.69 de
		N2	186.64 a	30.52 ef	149.48 c
		N3	185.27 a	27.38 fgh	176.38 b
		N4	188.05 a	24.56 h	214.23 a
	W2	N1	139.71 b	34.2 d	110.06 ef
		N2	142.82 b	32.01 de	126.93 d
		N3	142.48 b	29.76 efg	146.36 c
		N4	141.89 b	28.54 fg	186.1 b
	W3	N1	97.69 c	45.68 a	103.96 f
		N2	96.26 cd	45 ab	122.68 d
		N3	94.48 cd	42.01 bc	138.64 c
		N4	92.99 d	41.78 c	179.65 b
	Significant level (F value)				
	W		8248.510 ***	261.136 ***	58.293 ***
	N		0.697 ns	12.968 ***	252.141 ***
	W × N		3.083 *	1.958 ns	2.689 *

Note: Different lowercase letters in the same column indicate significant differences between different treatments, * indicates a significance level of *p* < 0.05, ** indicates a significance level of *p* < 0.01, *** indicates a significance level of *p* < 0.001, and ns indicates no significant effect (*p* > 0.05).

**Table 3:** Effects of different water and nitrogen treatments on cucumber quality indices in spring and autumn.

Season	Treatment		TSS (%)	SP (mg·g^−1^)	SSC (mg·g^−1^)	OA (%)	VC (mg·100 g^−1^)	FW (%)
Spring	W1	N1	3.31 bc	1.51 ab	12.18 bcde	0.133 a	6.35 a	96.87 abcd
		N2	3.37 abc	1.58 a	12.45 bcde	0.128 a	6.72 a	96.35 ef
		N3	3.3 bc	1.65 a	11.40 bcd	0.129 a	6.75 a	96.48 cdef
		N4	3.24 c	1.54 a	11.30 cde	0.109 a	7.39 a	96.71 bcde
	W2	N1	3.39 abc	1.16 b	12.68 abc	0.13 a	7.19 a	97.16 ab
		N2	3.41 abc	1.51 ab	12.49 bcd	0.128 a	6.80 a	96.42 def
		N3	3.37 abc	1.61 a	12.22 bcde	0.119 a	6.92 a	96.54 cdef
		N4	3.29 bc	1.42 a	11.88 cde	0.117 a	7.02 a	97.30 a
	W3	N1	3.51 ab	1.13 b	13.69 a	0.123 a	8.66 a	96.94 abc
		N2	3.53 a	1.47 ab	13.22 ab	0.127 a	6.93 a	96.15 f
		N3	3.42 abc	1.55 a	12.6 abc	0.118 a	7.10 a	96.37 ef
		N4	3.43 abc	1.36 a	12.51 bcd	0.107 a	6.61 a	96.87 abcd
	Significant level (F value)							
	W		5.024 **	4.334 *	10.966 ***	1.407 ns	0.188 ns	4.332 *
	N		1.234 ns	7.222 ***	5.366 **	3.852 *	0.128 ns	17.100 ***
	W × N		0.084 ns	1.095 ns	0.404 ns	0.379 ns	0.282 ns	0.834 ns
Autumn	W1	N1	3.23 bc	1.09 a	10.62 abc	0.144 a	6.87 ab	96.55 b
		N2	3.24 abc	1.14 a	11.12 abc	0.135 ab	7.44 ab	96.52 b
		N3	3.29 abc	1.13 a	10.21 abc	0.134 ab	7.51 ab	96.6 b
		N4	3.21 c	1.03 a	9.69 c	0.124 bc	7.57 ab	96.54 b
	W2	N1	3.27 abc	0.98 a	11.5 ab	0.145 a	4.45 c	96.58 b
		N2	3.34 ab	1.03 a	11.55 ab	0.126 bc	5.62 bc	96.62 b
		N3	3.31 abc	1.01 a	11.59 ab	0.120 bc	6.34 ab	97.05 a
		N4	3.24 abc	0.94 a	11.53 ab	0.120 bc	8.32 a	96.57 b
	W3	N1	3.29 abc	0.95 a	11.82 ab	0.118 bc	6.83 ab	96.54 b
		N2	3.40 a	0.95 a	12.04 a	0.112 c	7.08 ab	96.6 b
		N3	3.40 a	0.91 a	12.05 a	0.112 c	7.25 ab	96.6 b
		N4	3.32 abc	0.87 a	11.66 ab	0.109 c	8.3 a	96.56 b
	Significant level (F value)							
	W		5.566 **	2.713 ns	8.578 ***	13.352 ***	2.995 ns	3.356 *
	N		2.438 ns	0.220 ns	1.222 ns	5.288 **	5.320 **	3.066 *
	W × N		0.317 ns	0.077 ns	0.488 ns	0.682 ns	1.398 ns	1.728 ns

Note: TSS stands for total soluble solids, SP stands for soluble protein, SSC stands for soluble sugar content, OA stands for organic acids, VC stands for vitamin C, and FW stands for fruit water content. Different lowercase letters in the same column indicate significant differences between different treatments, * indicates a significance level of *p* < 0.05, ** indicates a significance level of *p* < 0.01, *** indicates a significance level of *p* < 0.001, and ns indicates no significant effect (*p* > 0.05).

**Table 4:** Factor loadings and variance contribution rates of the principal components.

Season	Spring				Autumn		
Indicator Variable		Factor Loading			Factor Loading		
		Primary Principal Component	Secondary Principal Component	Tertiary Principal Component	Primary Principal Component	Secondary Principal Component	Tertiary Principal Component
TSS (X_1_)		0.48219182	0.3967518	0.189213981	0.4865009	0.1917869	0.03291124
SP (X_2_)		−0.51315392	0.3227751	0.207072088	−0.493965	0.1208861	−0.24990705
SSC (X_3_)		0.54595202	0.2741942	0.000140492	0.4922473	0.2445882	0.26006091
OA (X_4_)		0.04177769	0.3755871	−0.867333974	−0.503199	0.324279	0.25802822
VC (X_5_)		0.43281668	−0.2354994	0.222485961	0.0721438	−0.752839	−0.34690726
FW (X_6_)		0.1304793	0.1304793	−0.345761443	0.1363613	0.4656798	−0.82577747
Characteristic value		1.6714629	1.2992702	0.972783	1.7055825	1.2073512	0.9407347
Variance contribution rate %		0.4656	0.2813	0.1577	0.4848	0.2429	0.1475
Cumulative contribution rate %		0.4656	0.747	0.9047	0.4848	0.7278	0.8753

**Table 5:** Comprehensive evaluation of cucumber quality parameters.

Spring			Autumn		
Treatment	Composite Score	Comprehensive Ranking	Treatment	Composite Score	Comprehensive Ranking
W1N1	−0.83136169	9	W1N1	−1.215605	10
W1N2	−0.02296137	7	W1N2	−1.046139	9
W1N3	−1.05153622	11	W1N3	−1.257684	11
W1N4	−1.17820078	12	W1N4	−1.511695	12
W2N1	0.17998933	5	W2N1	0.3250179	7
W2N2	0.21929423	4	W2N2	0.5685608	5
W2N3	−0.22360676	8	W2N3	0.6069301	4
W2N4	−1.01935963	10	W2N4	−0.216592	8
W3N1	1.92634157	1	W3N1	0.4915544	6
W3N2	1.40537137	2	W3N2	1.2355297	2
W3N3	0.42544815	3	W3N3	1.3533679	1
W3N4	0.17058179	6	W3N4	0.666753	3

**Table 6:** Correlation between soluble solid matter and soluble sugar content and quality indices of cucumber.

Season	Spring		Autumn	
Quality Index	TSS	SSC	TSS	SSC
TSS	-	0.93 ***	-	0.7 *
SP	−0.46	−0.63 *	0.5	−0.67 *
SSC	0.93 ***	-	0.7 *	-
SA	0.11	0.21	−0.62 *	−0.5
VC	0.35	0.48	−0.095	−0.18
FW	−0.3	−0.081	0.23	0.19
Absolute value accumulation	2.15	2.331	2.145	2.24

Note: The figures in the table are the correlation coefficients between the two quality indicators; * indicates a significance level of *p* < 0.05, and *** indicates a significance level of *p* < 0.001.

**Table 7:** Regression equations.

Season	Dependent Variable z	Regression Equation	R^2^	Combination of Treatments for z_max_		
				x (mm)	y (kg·ha^−1^)	z_max_
Spring	Yield (t·ha^−1^)	z = −102.71016 + 1.09285 W + 0.27054 N + 4.70428 × 10^−4^ W N − 0.00225 W^2^ − 5.05492 × 10^−4^ N^2^	0.98873 ***	285.03	401.14	106.61
	WUE (kg·m^−3^)	z =35.93815 + 0.03527 W + 0.07406 N + 1.22942 × 10^−4^ W N − 3.96169 × 10^−4^ W^2^ − 1.17448 × 10^−4^ N^2^	0.98866 ***	176.15	405.55	49.36
	PFP_n_ (kg·kg^−1^)	z = 68.25322 + 4.24433 W − 1.24084 N − 5.17234 × 10^−4^ W N − 0.00744 W^2^ + 9.11425 × 10^−4^ N^2^	0.99444 ***	278.23	216.00	416.70
	SSC (mg·g^−1^)	z = 14.25472 − 0.02364 W + 0.00776 N − 5.86699 × 10^−6^ W N + 3.23116 × 10^−5^ W^2^ − 2.39912 × 10^−6^ N^2^	0.9188 **	176.15	432.00	13.55
Autumn	Yield (t·ha^−1^)	z = 23.84722 − 0.16145 W + 0.1318 N − 1.04553 × 10^−4^ W N + 9.97985 × 10^−4^ W^2^ − 1.35042 × 10^−4^ N^2^	0.9374 **	189.50	414.37	52.31
	WUE (kg·m^−3^)	z = 77.41246 − 0.7036 W + 0.08085 N − 6.25667 × 10^−5^ W N + 0.00194 W^2^ − 7.94015 × 10^−5^ N^2^	0.97833 ***	94.75	432.00	45.74
	PFP_n_ (kg·kg^−1^)	z = 320.48707 − 0.33575 W − 0.73897 N − 0.00112 W N + 0.0035 W^2^ + 8.07537 × 10^−4^ N^2^	0.98804 ***	189.50	216.00	214.62
	SSC (mg·g^−1^)	z = 9.07809 + 0.01858 W + 0.01315 N + 4.70747 × 10^−5^ W N − 1.73988 × 10^−4^ W^2^ − 2.77265 × 10^−5^ N^2^	0.92809 **	96.68	317.39	12.07

Note: x and y represent the amount of irrigation and nitrogen application, respectively. ** indicates a significance level of *p* < 0.01, and *** indicates a significance level of *p* < 0.001.

**Table 8:** Greenhouse cucumber irrigation treatments (mm).

Irrigation Treatment	Spring	Autumn
W1	287.3	189.5
W2	231.73	142.13
W3	176.15	94.75

**Table 9:** Greenhouse cucumber fertigation treatments (kg·ha^−1^).

Season	Fertilization Treatment	Base Fertilizer				Each Fertilizer Amount			Total Amount of Fertilizer		
		Organic Fertilizer	N	P_2_O_5_	K_2_O	N	P_2_O_5_	K_2_O	N	P_2_O_5_	K_2_O
Spring	N1	9.1 × 10^3^	129.6	120	108	60.48	-	50.4	432	120	360
	N2		108	120	108	50.4	-	50.4	360	120	360
	N3		86.4	120	108	40.32	-	50.4	288	120	360
	N4		64.8	120	108	30.24	-	50.4	216	120	360
Autumn	N1	9.1 × 10^3^	86.4	120	72	69.12	-	57.6	432	120	360
	N2		72	120	72	57.6	-	57.6	360	120	360
	N3		57.6	120	72	46.08	-	57.6	288	120	360
	N4		43.2	120	72	34.56	-	57.6	216	120	360

## Data Availability

The raw data supporting the conclusions of this article will be made available by the authors, without undue reservation. The data are not publicly available due to copyright.

## Abbreviations

The following abbreviations are used in this manuscript:

DW	Dry weight
ET	Crop evapotranspiration
FW	Fruit water content
*LAI*	Leaf area index
OA	Organic acid
PCA	Principal component analysis
PFP_n_	Partial factor productivity of nitrogen
SP	Soluble protein
SSC	Soluble sugar content
TSS	Total soluble solids
VC	Vitamin C
WUE	Water use efficiency
Y_a_	Economic yield of cucumber
